# Socio-Economic Differences in Cardiovascular Health: Findings from a Cross-Sectional Study in a Middle-Income Country

**DOI:** 10.1371/journal.pone.0141731

**Published:** 2015-10-29

**Authors:** Janko Janković, Miloš Erić, Dragana Stojisavljević, Jelena Marinković, Slavenka Janković

**Affiliations:** 1 Institute of Social Medicine, Faculty of Medicine, University of Belgrade, Belgrade, Serbia; 2 Center for European Integration and Public Management, Faculty of Economics, Finance and Administration, Singidunum University, Belgrade, Serbia; 3 Institute of Public Health, Banja Luka, Republic of Srpska, Bosnia and Herzegovina; 4 Institute of Medical Statistics and Informatics, Faculty of Medicine, University of Belgrade, Belgrade, Serbia; 5 Institute of Epidemiology, Faculty of Medicine, University of Belgrade, Belgrade, Serbia; Hunter College, UNITED STATES

## Abstract

**Background:**

A relatively consistent body of literature, mainly from high-income countries, supports an inverse association between socio-economic status (SES) and risk of cardiovascular disease (CVD). Data from low- and middle-income countries are scarce. This study explores SES differences in cardiovascular health (CVH) in the Republic of Srpska (RS), Bosnia and Herzegovina, a middle-income country.

**Methods:**

We collected information on SES (education, employment status and household’s relative economic status, i.e. household wealth) and the 7 ideal CVH components (smoking status, body mass index, physical activity, diet, blood pressure, total cholesterol, and fasting blood glucose) among 3601 participants 25 years of age and older, from the 2010 National Health Survey in the RS. Based on the sum of all 7 CVH components an overall CVH score (CVHS) was calculated ranging from 0 (all CVH components at poor levels) to 14 (all CVH components at ideal levels). To assess the differences between groups the chi-square test, t-test and ANOVA were used where appropriate. The association between SES and CVHS was analysed with multivariate linear regression analyses. The dependent variable was CVHS, while independent variables were educational level, employment status and wealth index.

**Results:**

According to multiple linear regression analysis CVHS was independently associated with education attainment and employment status. Participants with higher educational attainment and those economically active had higher CVHS (b = 0.57; CI = 0.29–0.85 and b = 0.27; CI = 0.10–0.44 respectively) after adjustment for sex, age group, type of settlement, and marital status. We failed to find any statistically significant difference between the wealth index and CVHS.

**Conclusion:**

This study presents the novel information, since CVHS generated from the individual CVH components was not compared by socio-economic status till now. Our finding that the higher overall CVHS was independently associated with a higher education attainment and those economically active supports the importance of reducing socio-economic inequalities in CVH in RS.

## Introduction

The growing body of literature shows that socio-economic status (SES) is strongly associated with risk of cardiovascular diseases (CVD), particularly in high income countries where people with low SES have a greater risk of developing CVD [1–4]. This issue remains largely unexplored in low- and middle-income countries, which bear over 80% of the world’s burden of CVD [5], but evidence is emerging that the increasing wealth of these countries leads to replication of the patterns seen in high-income countries [1,6].

The inverse association between SES and CVD risk in high-income countries is the result of the high prevalence and combining effects of multiple behavioral and psychosocial risk factors among worse-off [1]. Recently, Franks et al. [4] found that accounting for changes in key traditional CVD risk factors and anti-hypertensive medication explained little of the independent effect of SES on coronary heart disease (CHD) risk and that SES does not appear to be simply a proxy for poor health-care access or poor adherence to treatments such as smoking cessation or medication.

Republic of Srpska (RS) is one of two autonomous entities that constitute Bosnia and Herzegovina (BH), a country in Southeastern Europe, located in the Balkan Peninsula. Major demographic, socio-economic, political and health care system changes, especially from 1995 and onward, significantly influenced the lives and working conditions of the population and, consequently, their health status. With a per capita gross national income of US$ 4,780 in 2013 [7], BH ranks as an upper middle-income country [8]. The unemployment rate in 2010 was around 27% [9], while poverty was estimated to be 17.9% in 2011 [7]. BH’s overall social indicators are high with a human development index of 0.726 in 2010, and value of gender development index that suggests some gender inequality, particularly in economic activities [10].

Recently, the American Heart Association (AHA) proposed the concept of ideal cardiovascular health (CVH), which is defined as the simultaneous presence of 7 ideal CVH components: 4 favorable behaviors (nonsmoking, ideal body mass index, physical activity at goal level, and dietary patterns consistent with current guideline recommendations) and 3 favorable health factors (ideal levels of total cholesterol, blood pressure, and fasting glucose) [11]. A number of researchers have used this construct to investigate the relationship between ideal CVH and CVD. Achieving a greater number of ideal health metrics is associated with a lower risk of cardiovascular events [12–14] and CVD mortality [15–17].

The aim of this study was to determine distribution of CVH components, overall CVHS and CVHS categories by measures of socio-economic status in the RS, BH, a middle-income European country, employing the concept of ideal CVH proposed by AHA.

## Method

### Study design and sample

This cross-sectional study utilized data collected in the 2010 National Health Survey (NHS) in RS, BH. Methodology, ethics consideration and quality control of the survey data have been detailed elsewhere [18,19]. In brief, 4673 adults aged ≥18 years have been identified in the randomly selecting households, out of which 4170 were interviewed yielding a response rate of 89.2%. All persons who signed the informed consent form underwent physical examinations (anthropometric and blood pressure measurements, and blood tests) at home by trained staff. Information on socio-demographic, and lifestyle factors was collected using standardized questionnaires.

The study was approved by the Ethics Committee of the Public Health Institute of RS.

In the previously published manuscripts related to CVH of RS participants [18,20] we used the sample of all adults participants aged ≥18 years (n = 4170) while for the purpose of the present study only participants aged ≥25 years (as suggested in the literature for studies investigating education) with complete information on all study variables (n = 3601), were included in the study. Besides the different age, consequently different number of participants and different aims, in the present study we used new variables, such as the Cardiovascular Health Score (CVHS), and Demographic and Health Survey Wealth Index (DHS Wealth Index).

### Study variables

Demographic and socio-economic characteristics (age, sex, type of settlement, marital status, education, and employment status) were self-reported. Type of settlement was identified at the survey level as urban or rural. Marital status was categorized in two groups: married/living with partner or living without partner.

Educational attainment was categorized as low (no schooling, incomplete primary school and primary school), middle (three or four years of secondary education), and high (college and university education).

According to the International Labour Organisation (ILO) guidelines [21] employment status was defined by one of two groups: economically active [those in employment plus ILO unemployed (those who are without a job, are available to start work in the next two weeks, who have been seeking a job in the last four weeks or are waiting to start a job already obtained) and economically inactive (people who are neither in employment or unemployment—this includes those looking after a home or family, retired, full-time students, long term sick or disabled)].

Besides educational level and employment status the DHS Wealth Index (hereafter wealth index) was used as a measure of socio-economic status [22]. The wealth index was calculated using easy-to-collect data on a household's ownership of selected assets, such as television, cellular phone, refrigerator, computer, washing machine, air conditioning, central heating, car; materials used for housing construction; types of water access and sanitation facilities etc. Statistical procedure principal components analysis (PCA) was used to assign the weights or factor scores to each variable as described in detail elsewhere [23]. According to the wealth index respondents were classified into five socio-economic groups or quintiles: the poorest, poorer, middle class, richer and the richest class. For the purpose of analyses they were classified into three socio-economic groups: poorest-poor group, middle class group, and richer-richest group.

Age, sex, marital status and settlement were selected as potential confounders according to the literature data [24,25].

### Cardiovascular Health Score (CVHS)

Each of the 7 CVH metric (smoking, BMI, physical activity, healthy diet score, total cholesterol, blood pressure, and fasting glucose) was given a score of 0, 1, or 2 points, representing poor, intermediate, or ideal cardiovascular health, respectively, as predefined categories (Table 1).

**Table 1:** Cardiovascular health—definition* and categories points.

Health metric	Level / Categories points		
	Poor / 0	Intermediate / 1	Ideal / 2
Smoking	Current	Former, quit ≤12 months	Never or quit >12 months
Body mass index	≥30 kg/m^2^	25–29.99 kg/m^2^	<25 kg/m^2^
Physical activity	Inactive	Moderately active	Active
Healthy diet score	<21	21–25	≥26
Total cholesterol	≥240 mg/dL	200–239 mg/dL or treated to goal	<200 mg/dL, untreated
Blood pressure	SBP ≥140 or DBP ≥90 mm Hg	SBP 120–139 or DBP 80–89 mm Hg or treated to goal	SBP/DBP <120/80 mm Hg, untreated
Fasting glucose	≥126 mg/dL	100–125 mg/dL or treated to goal	<100 mg/dL, untreated

*According to the American Heart Association except for healthy diet score, and physical activity.

DBP—diastolic blood pressure; SBP—systolic blood pressure.

Based on the sum of all 7 CVH metrics an overall CVHS was calculated ranging from 0 (all CVH metrics at poor levels) to 14 (all CVH metrics at ideal levels), and for the purpose of describing prevalence estimates categorized into low (0–4), medium (5–9) or high (10–14) CVH category, according to the literature [26,27].

**Fig 1:**
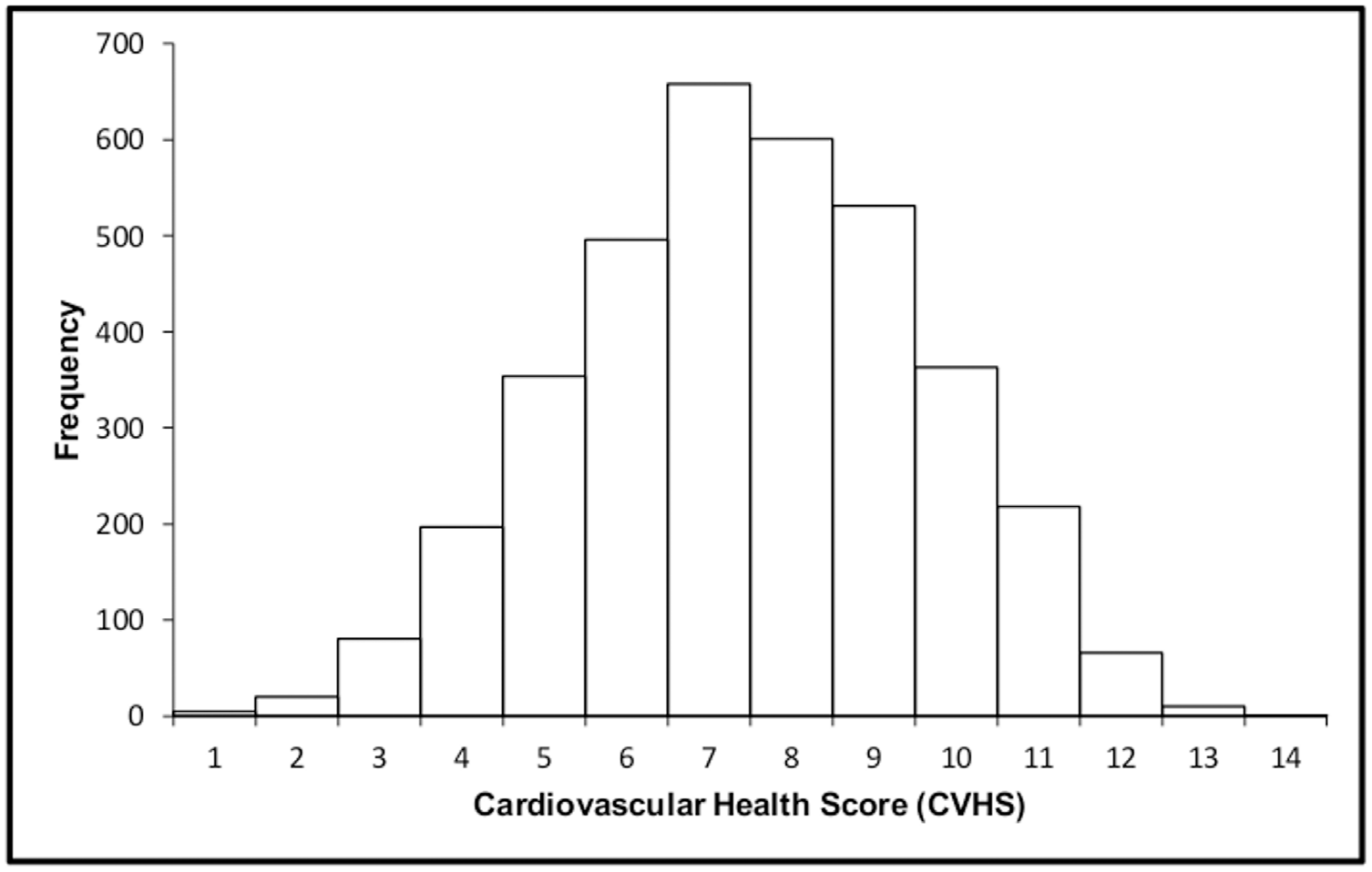
The distribution of Cardiovascular Health Score in survey participants. Smoking status (current, former, or never smokers) was derived from interviews. Physical activity as described previously [18] was self-reported. Those who participated in physical activity four times or more a week for at least 30 minutes were categorized as physically active, those who exercised less than four times a week but at least 2–3 times a month were categorized as moderately active and those who exercised several times a year or did not exercise at all were categorized as physically inactive.

The distribution of the overall CVHS score showed a bell-shaped curve with mean score of 7.5 points and almost negligible number of participants with the best (13–14 points) or the worst values (0–1 points) (Fig 1).

As described in detail elsewhere [20] for the purpose of assessment of dietary intake the healthy diet score (HDS) had been created. We used the data from self-administered food frequency questionnaire and food habits questionnaire. The total HDS was the sum of 11 indicators identified for each dietary guideline for adults in the RS. The HDS had a possible range from 0 to 38 points, with a higher score reflecting increased compliance with the dietary guidelines.

Height and weight were measured with participants wearing light clothing and no shoes, by trained health professionals. A portable electronic medical scale (Seca, 877) was used to measure body weight to the nearest half-kilogram. Standing body height (cm) was measured to the nearest 0.1 cm with a portable wall-mounted stadiometer (Seca, 206). Body mass index (BMI) was calculated as weight divided by height squared (kg/m^2^).

Systolic blood pressure (SBP, mm Hg) and diastolic blood pressure (DBP, mmHg) were measured using mercury sphygmomanometer—diplomat-presameter (Riester, CE 0124, Germany) with appropriately sized cuffs, after the participants have been resting in a sitting position for at least 10 minutes. Sitting blood pressure was measured three times after a 5-minute rest. The mean of the last two measurements was used for analysis.

To measure total cholesterol (TC, mg/dL) and fasting blood glucose (FG, mg/dL), overnight capillary blood samples were obtained and analysed using a calibrated Accutrend^®^ Plus GCTL analyser (Roche Diagnostics, Mannheim, Germany). Use of antihypertensive, cholesterol-lowering, and glucose-lowering medications within the past two weeks of baseline interview was self-reported.

### Statistical analysis

The main outcome was CVHS treated both as a categorical variable for the distribution of prevalence estimates and as a continuous variable in the association analyses. The main exposure variables—education, employment status and wealth index were treated as categorical variables. Continuous variables were described with means and standard deviations while categorical ones with frequencies and percentages. In order to assess the differences between groups the chi-square test, t-test and ANOVA were used where appropriate.

The association between SES and CVHS was analysed with univariate and multivariate linear regression analyses. The dependent variable was CVHS (as continuous variable from 0 through 14), while independent variables were the main exposure variables (all categorical): educational level (low, middle and high), employment status (economically active and economically inactive), and wealth index (poorest and poor, middle, richer and richest), and the adjustment variables (all categorical): sex, age group (young, middle-aged and old), type of settlement (urban or rural), and marital status (married/living with partner or living without partner). We defined and analysed the following models: three unadjusted univariate models for each SES variable, unadjusted multivariate model with all SES variables, three multivariate models standardized on age and sex for each SES variable, multivariate model standardized on age, sex and all SES variables, multivariate model standardized on age, sex, type of settlement, marital status and all SES variables, and finally multivariate model which included all previously mentioned variables and all interactions between age, sex and SES variables. They are presented with partial coefficients of regression *b* and their 95% confidence interval. The analyses were restricted to participants with complete information on all variables (3601). We excluded 380 participants under 25 years of age and 189 participants with missing information (i.e. those with incomplete interview or physical examination) on any variable included in the analysis.

To see if there was any difference in the models using all the AHA variables versus models which excluded the diet and physical activity variables which were derived differently according to available data for RS, we performed sensitivity analyses.

All statistical analyses were performed using SPSS version 20.0 software (SPSS Inc., Chicago, IL, USA) and STATA version 11.1 (StataCorp LP College Station, TX, USA) with the complex sampling design taken into account. Statistical significance was set at 2-sided *P* <0.05.

## Results

The present study included 1987 (55%) women and 1614 (45%) men, in total 3601 adults aged ≥25 years. Their mean age was 52.9 years. Most of the participants were rural dwellers (59.3%), married or living with partner (70.6%), had middle education (47.3) and belonged to the poorest and poor group (40.6%). Out of all participants 30.5% were employed, 22.6% unemployed, 23.2% were retired, 21.3% were housewives and 2.4% were others (students and disabled people).

Distribution of characteristics of study participants by sex and CVHS categories was presented in Table 2.

**Table 2:** Distribution of characteristics of study participants (n = 3601) by sex and cardiovascular health score (CVHS) categories.

Characteristics	All participants (n = 3601)	By sex			By CVHS categories			
		Males (n = 1614)	Females (n = 1987)	*P* *	0–4 (n = 303)	5–9 (n = 2640)	10–14 (n = 658)	*P* *
Age, n (%)								
25–44	1167	550 (47.1)	617 (52.9)	<0.001	37 (3.2)	767 (65.7)	363 (31.1)	<0.001
45–64	1524	710 (46.6)	814 (53.4)		167 (11.0)	1144 (75.1)	213 (14.0)	
≥65	910	354 (38.9)	556 (61.1)		99 (10.9)	729 (80.1)	82 (9.0)	
Settlement, n (%)								
Urban	1467	635 (43.3)	832 (56.7)	ns.	123 (8.4)	1061 (72.3)	283 (19.3)	ns.
Rural	2134	979 (45.9)	1155 (54.1)		180 (8.4)	1579 (74.0)	375 (17.6)	
Marital status, n (%)				<0.001				
Married/with partner	2541	1209 (47.6)	1332 (52.4)		220 (8.7)	1860 (73.2)	461 (18.1)	ns.
Without partner	1060	405 (38.2)	655 (61.8)		83 (7.8)	780 (73.6)	197 (18.6)	
Education, n (%)								
Low	1600	517 (32.3)	1083 (67.7)	<0.001	169 (10.6)	1214 (75.9)	217 (13.6)	<0.001
Middle	1704	934 (54.8)	770 (45.2)		119 (7.0)	1230 (72.2)	355 (20.8)	
High	297	163 (54.9)	134 (45.1)		15 (5.1)	196 (66.0)	86 (29.0)	
Employment status, n (%)								
Economically active	1912	1093 (57.2)	819 (42.8)	<0.001	123 (6.4)	1355 (70.9)	434 (22.7)	<0.001
Economically inactive	1689	521 (30.8)	1168 (69.2)		180 (10.7)	1285 (76.1)	224 (13.3)	
Wealth index, n (%)								
Poorest and poor	1463	597 (40.8)	866 (59.2)	<0.001	145 (9.9)	1085 (74.2)	233 (15.9)	0.006
Middle	715	304 (42.5)	411 (57.5)		55 (7.7)	519 (72.6)	141 (19.7)	
Richer and richest	1423	713 (50.1)	710 (49.9)		103 (7.2)	1036 (72.8)	284 (20.0)	
SBP, mean±SD	137±21	138±19	137±23	n.s.	153±19	139±21	124±16	<0.001
DBP, mean±SD	85±11	86±10	84±11	<0.001	93±10	86±11	79±9	<0.001
BMI, mean±SD	26.9±4.9	26.9±4.3	26.9±5.3	n.s.	31.7±4.9	27.1±4.7	23.8±3.3	<0.001
Cholesterol, mean±SD	5.24±1.32	5.1±1.29	5.33±1.34	<0.001	6.21±1.08	5.32±1.28	4.47±1.18	<0.001
Glucose, mean±SD	5.05±1.71	5.0±1.67	5.05±1.75	n.s.	6.54±2.42	5.03±1.65	4.47±1.04	<0.001
CVHS, mean ±SD	7.49±2.13	7.31±2.08	7.63±2.15	<0.001	3.55±0.69	7.17±1.31	10.58±0.74	<0.001

Economically active—employed and unemployed; Economically inactive—people looking after a home or family, retired, students and disabled. ns.—not significant

*According to chi-square, t-test or ANOVA where appropriate.

Females were older, more frequently lived without partner, were less educated, more frequently economically inactive and more frequently had poor and middle wealth index. Men had higher levels of DBP, lower levels of cholesterol and lower values of CVHS.

The inadequate / low CVHS category (values of CVHS from 0 to 4) had 8.4% of participants, the average / medium CVHS category (values from 5 to 9) was present in 73.3% of participants while the optimal / high CVHS category (values from 10 to14) had 18.3% of all participants. People in the youngest age group (25–44) more frequently belonged to the optimal CVHS category in comparison to the middle (45–64) and the oldest (≥65) age group. The prevalence of people with high / optimal CVHS was significantly higher in those with high education, in economically active and in the better-off in comparison to the low educated economically inactive and poorest and poor people respectively. There were no differences in the type of settlement (urban or rural) and marital status between people with low, medium and high CVHS. The levels of BMI and health factors (SBP, DBP, TC and FG) decreased with the increase of CVHS and were the lowest in the group of participants with the highest CVHS (Table 2).

The prevalence of ideal CVH metrics ranged from 4.8% for HDS to 66.7% for the FG level (Table 3).

**Table 3:** Prevalence (95% CI)* of cardiovascular health (CVH) components by cardiovascular health score (CVHS) categories.

CVH components	All participants (n = 3601)	CVHS categories			
		CVHS = 0–4 (n = 303)	CVHS = 5–9 (n = 2640)	CVHS = 10–14 (n = 658)	*P* †
Smoking					
Current	32.4 (30.3–34.4)	58.4 (53.5–63.3)	35.5 (33.8–37.1)	3.2 (0.1–6.6)	<0.001
Former, quit ≤12 months	16.8 (15.1–18.5)	21.0 (16.8–25.1)	17.1 (15.7–18.5)	12.3 (9.5–15.2)	
Never or quit >12 months	50.8 (48.7–53.0)	20.6 (15.5–25.8)	47.4 (45.7–49.2)	84.4 (80.9–88.0)	
Body mass index					
≥30 kg/m2	30.1 (28.3–32.0)	65.6 (61.1–70.0)	23.3 (21.8–24.8)	1.5 (0.0–4.6)	<0.001
25–29.99 kg/m^2^	32.8 (30.5–35.0)	27.5 (23.8–31.4)	43.1 (41.3–45.0)	27.5 (22.1–33.0)	
<25 kg/m^2^	37.1 (35.0–39.2)	6.9 (1.8–12.0)	33.5 (31.8–35.3)	70.9 (67.4–74.4)	
Physical activity					
Inactive	43.4 (41.3–45.5)	71.0 (66.0–76.0)	42.2 (40.5–43.9)	16.9 (13.5–20.4)	<0.001
Moderately active	17.2 (15.4–19.0)	16.7 (12.3–21.1)	21.2 (19.7–22.7)	13.7 (10.6–16.8)	
Active	39.4 (37.3–41.6)	12.3 (7.1–17.4)	36.6 (34.9–38.4)	69.4 (65.8–73.0)	
Healthy diet score					
Poor <21	59.4 (57.2–61.6)	77.4 (72.2–82.6)	62.9 (61.1–64.6)	38.0 (34.4–41.6)	<0.001
Intermediate 21–25	35.8 (33.6–37.9)	21.7 (16.4–26.9)	33.5 (31.7–35.2)	52.2 (48.6–55.8)	
Ideal ≥26	4.8 (3.8–5.7)	1.0 (0.0–3.2)	3.6 (2.8–4.4)	9.8 (8.2–11.4)	
Total cholesterol					
≥240 mg/dL	28.3 (26.5–30.2)	58.4 (53.9–62.8)	23.6 (22.0–25.1)	3.1 (0.0–6.2)	<0.001
200–239 mg/dL or treated to goal	30.5 (28.3–32.7)	30.0 (24.6–35.3)	38.4 (36.6–40.2)	23.2 (19.5–26.9)	
<200 mg/dL, untreated	41.2 (39.0–43.3)	11.7 (6.5–16.8)	38.1 (36.4–39.8)	73.7 (70.1–77.3)	
Blood pressure					
SBP ≥140 or DBP ≥90 mm Hg	36.7 (34.7–38.7)	65.1 (60.3–69.9)	32.5 (30.9–34.1)	12.5 (9.1–15.8)	<0.001
SBP 120–139 or DBP 80–89 mm Hg or treated	49.8 (47.5–52.1)	30.5 (25.0–36.0)	57.5 (55.7–59.4)	61.5 (57.6–65.3)	
SBP/DBP <120/80 mm Hg, untreated	13.5 (12.0–14.9)	4.4 (1.0–7.9)	9.9 (8.8–11.1)	26.1 (23.7–28.5)	
Fasting glucose					
≥126 mg/dL	13.0 (11.9–14.1)	31.5 (28.8–34.2)	5.2 (4.3–6.1)	2.4 (0.1–4.2)	<0.001
100–125 mg/dL or treated to goal	20.3 (18.5–22.1)	33.9 (29.6–38.2)	19.7 (18.3–21.2)	7.4 (4.3–10.4)	
<100 mg/dL, untreated	66.7 (64.7–68.6)	34.6 (30.0–39.2)	75.1 (73.5–76.6)	90.3 (87.1–93.5)	

*Adjusted by sex, age, education, employment status and wealth index.

^†^According to chi-square test.

Most participants had ideal levels of smoking status (never or quit >12 months), BMI (<25 kg/m2), TC (<200 mg/dL, untreated) and FG (<100 mg/dL, untreated). The prevalence of ideal CVH components for smoking, physical activity, TC and FG was the highest in participants with high CVHS (10–14). For HDS and SBP this was true for those who had poor or medium CVHS respectively.

Table 4 shows the estimated changes in mean CVHS by SES indicators in unadjusted models and models variously standardized by age and sex only (model A); by age, sex and SES indicators (model B); by SES indicators, type of settlement and marital status (model C); by SES indicators type of settlement, marital status and all interactions with sex and age. The independent effect of age explains the most part of variability in CVHS, but its variability is also explained by the independent effects of sex, education and employment.

**Table 4:** Change in mean CVHS among health survey participants by SES indicators in models variously standardized.

Characteristics	Unadjusted results − ULR model	Unadjusted results − MLR model	Model A − standardised by age and sex only	Model B = Model A + additionally standardised by SES indicators: education (when looking at employment status and wealth index), employment status (when looking at education and wealth) and wealth (when looking at education and employment status)	Model C = Model A + additionally standardised by SES indicators, type of settlement and marital status	Model D = Model A + additionally standardised by SES indicators, type of settlement and marital status + all interactions with sex and age*
Education						
Low	Reference	Reference	Reference	Reference	Reference	Reference
Middle	**0.59 (0.44 to 0.73)**	**0.32 (0.16 to 0.48)**	**0.25 (0.10 to 0.41)**	**0.21 (0.05 to 0.38)**	**0.25 (0.08 to 0.42)**	**0.26 (0.07 to 0.43)**
High	**0.89 (0.63 to 1.15)**	**0.57 (0.30 to 0.85)**	**0.57 (0.30 to 0.83)**	**0.51 (0.24 to 0.78)**	**0.57 (0.29 to 0.85)**	**0.46 (0.09 to 0.82)**
Employment status						
Economically active	**0.76 (0.62 to 0.89)**	**0.58 (0.43 to 0.73)**	**0.33 (0.16 to 0.50)**	**0.27 (0.09 to 0.44)**	**0.27 (0.10 to 0.44)**	**0.20 (0.01 to 0.39)**
Economically inactive	Reference	Reference	Reference	Reference	Reference	Reference
Wealth Index						
Poorest and poor	Reference	Reference	Reference	Reference	Reference	Reference
Middle	**0.28 (0.09 to 0.47)**	0.11 (-0.07 to 0.30)	0.11 (-0.07 to 0.29)	0.05 (-0.12 to 0.24)	0.06 (-0.11 to 0.25)	0.08 (-0.10 to 0.26)
Richer and richest	**0.33 (0.17 to 0.48)**	0.04 (-0.12 to 0.20)	0.08 (-0.06 to 0.24)	-0.02 (-0.17 to 0.14)	0.01 (-0.15 to 0.16)	0.02 (-0.13 to 0.19)

CVHS—Cardiovascular health score; SES—socio-economic status; ULR—Univariate linear regression; MLR—Multivariate linear regression. Economically active − employed and unemployed); Economically inactive—people looking after a home or family, retired, students and disabled.

*additionally significant: Middle age x High educational level (-0.12 (-0.20 to -0.04)); Middle age x Economically active (0.08 (0.06 to 0.27)); Female x Middle educational level (0.14 (0.06 to 0.22)) and Female x High educational level (0.08 (0.01 to 0.16)).

According to multiple linear regression analysis, higher CVHS was independently associated with higher education attainment, and economically active status of participants. We did not find any statistically significant association between the wealth index and CVHS (Table 4). There were also evidences on significant interactions from the last generated model. Higher CVHS was seen in middle-aged and economically active participants compared to young and those economically inactive respectively; and in middle and high educated women towards low educated men. Middle-aged, high educated participants had lower CVHS in comparison to those young with low education (Table 4).

The results of sensitivity analyses were reported in S1 Table in the Supplementary Appendix. The correlation between CVHS based on all 7 CVH AHA components and newly constructed CVHS based on the 5 CVH AHA components (excluding healthy diet and physical activity variables) was high (r = 0.871; p<0.001). There were differences between two sets of models concerning the independent association of employment status when interactions where entered in the model as well as some additional significant interactions.

## Discussion

Our results have shown substantial socio-economic differences in CVH for two out of three measures of SES used in the study: education attainment and employment status.

A positive high independent association was found between level of education and CVHS, i.e. the proportion of people with better CVHS was significantly higher in those with a higher level of education. This is in accordance with the results from several studies where participants with high educational attainment had better CVH status in comparison with those with low education. In two population-based cross-sectional studies performed in the US [28,29] the probability of optimal CVH was found to be greater among the college educated or above. According to the Spanish study [30], people with secondary or higher education had better CVH. Data from six cross-sectional studies conducted in Denmark [31] from 1978 to 2006 indicated an increasing trend in ideal CVH with a more favorable risk profile among persons with high educational level. Differences in risk factor levels across education groups in the Tromsø Study in 1994–1995 (n = 22108) and in 2007–2008 (n = 11565) were persistent for all cardiovascular risk factors, except for cholesterol, over time, with a more unfavorable pattern in the lowest education group [32]. In the large cohort of female health professionals followed up over a 10-year period (known as Women’s Health Study), Albert et al. observed a progressive decrease in incident CVD events with increasing levels of graduate education. This relationship was explained only partially by traditional and novel risk factors for CVD [33].

In contrast to higher income countries, higher attained educational level may not be protective against cardiovascular events in low- and middle-income countries, particularly in women [2]. Rosengren et al. [34] investigated the effect of education and other measures of SES on risk of acute myocardial infarction (AMI) in patients and controls from 52 countries with diverse economic circumstances. They found that low education was the marker most consistently associated with increased risk for AMI, most clearly in high-income countries. In China, participants with the clustering of major CVD risk factors (two or more of the following: hypertension, diabetes, dyslipidemia and overweight) compared with participants without any defined CVD risk factor or with a single major CVD risk factor, were less educated [35]. These educational differences in CVH may be attributable to the fact that education reflects access to important health-related resources (personal, social, or structural) [36], and consequently higher education provides more coping skills for daily life issues that could negatively affect health (within the family, social, and work environment) and offers more opportunities to solve them [23]. In Iranian study education level was the strongest factor positively correlated with CVD risk factors, except for smoking in men [37]. Norwegian authors who investigated the relationship between obesity and education on more than 400,000 participants from 70 countries in the period 2002–2013, concluded that in low-income countries, obesity was more prevalent in individuals with higher education, while in medium-income and high-income countries, it shifts to be more prevalent among those with lower levels of education [38].

In this study we found that economically active people (both employed and unemployed) had better CVH in comparison to those out of the labour force (economically inactive people). This could be explained by the fact that in the economically inactive group in RS there is a considerable percentage of retired elderly people who are more frequently physically inactive and more often suffer from hypertension and elevated cholesterol level in comparison to those economically active. Another possible explanation is the increasing number of early retired people in RS due to permanent disability after the civil war in the 1990s. The presence of hypertension, diabetes, CVD [39] and unhealthy behavior, such as smoking [39,40] and obesity [39] was shown to predict early retirement risk due to health issues.

The second large group of economically inactive persons in our sample are housewives. It is generally assumed that female workers have better health than full-time homemakers, although this pattern was more consistent for women of low educational level [41–43]. In the Stockholm Female Coronary Risk Study the OR for CHD associated with being a housewife was 2.5 (95% CI: 1.3–4.7) as compared with women who had a white-collar occupation [44].

In the survey on Health Aging in most European countries, except in France, several lifestyle factors, most notably lack of physical activity and obesity, had statistically significant associations with non-participation in the labour force due to an early retirement, or being a homemaker [45].

In the current study, we failed to find any significant association between the wealth index and CVHS that is in line with some previous studies [46,47]. In the INTERHEART case-control study conducted in 52 countries, family income and numbers of possessions were only weakly or not at all independently related to CHD events [34].

It is well known that in low-income and middle-income countries links between SES and CVD and CVD risks are less consistent. Authors from India who performed a systematic review of 53 studies reported that, with the exception for smoking, CVD and risk factors (obesity, diabetes, elevated lipids and hypertension) are significantly more prevalent among better-off [48]. The possible explanation for increased prevalence of CVD in populations of high SES is that wealthy citizens in poor countries tend to have more behavioral risk factors than do poor citizens [1]. In contrast, authors from the neighboring Serbia [49] found a higher prevalence of CVD (myocardial infarction and stroke) and cardiovascular risk factors (hypertension, diabetes) in those who belonged to the poorest wealth index quintile category. The inverse relationship of income to incident CVD events, almost entirely attributable to traditional CVD risk factors, was also observed in above mentioned Women’s Health Study [33]. This inverse association between SES and CVD risk observed previously in high-income countries is the result of the high prevalence and combining effects of multiple behavioral and psychosocial risk factors among worse-off [1]. A possible explanation could also be the fact that the most disadvantaged groups have fewer material and social resources with which they can deal with their conditions.

To the best of our knowledge this study presents a novel information, since CVH and CVHS based on AHA criteria have not been compared previously by SES measured by three commonly used indicators—education, employment status and wealth.

We utilized a nationally representative population-based sample with extensive data on cardiovascular risk factors providing the metrics needed to calculate the CVHS.

However, several limitations of the study should be briefly stressed. Firstly, the cross-sectional study design prevents any conclusions regarding causality to be made. Secondly, information on several CVH components and socio-economic variables has been obtained through a self-administered questionnaire, which may be subject to recall bias or selective reporting. Thirdly, we were unable to study associations between CVHS and CVD outcomes.

Notwithstanding all previously mentioned limitations, our study shows that socio-economic differences in CVH exist in the population of RS. Our findings revealed that participants with low educational attainment and those economically inactive had worse CVH status. Therefore, comprehensive and targeted prevention strategies and interventions at both individual and population-based levels aimed in improving CVH status and reducing CVH differences must be considered. Sustained and focused efforts among all SES groups with primary focus on the most disadvantaged are urgently needed.

## Supporting Information

S1 Table Change in mean CVHS based on AHA components only, without diet and physical activity among participants by SES indicators in models variously standardized. (DOCX)
