# Supplementary material for: Socio-Economic Differences in Cardiovascular Health: Findings from a Cross-Sectional Study in a Middle-Income Country
Source: PLoS One. 2015 Oct 29;10(10):e0141731. doi: 10.1371/journal.pone.0141731 (PMC4626110; doi:10.1371/journal.pone.0141731)
Supplement: S1 Table — (DOCX) [file pone.0141731.s001.docx]

**S1 Table. Change in mean CVHS based on AHA components (5) only, without diet and physical activity among participants by SES indicators in models variously standardised**

|  | Unadjusted results  ULR model | Unadjusted results  MLR model | Model A − standardised by age categories and sex only | Model B = Model A + additionally standardised by other SES indicators; education (when looking at employment status and wealth index), employment (when looking at education and wealth) and for wealth (when looking at education and employment) | Model C = Model A+ additionally standardised by all SES indicators, type of settlement and marital status | Model D = Model A + additionally standardised by all SES indicators, type of settlement and marital status + interactions with sex and age* |
| --- | --- | --- | --- | --- | --- | --- |
| Education |  |  |  |  |  |  |
| Low | Reference | Reference | Reference | Reference | Reference | Reference |
| Middle | **0.42 (0.29 to 0.54)** | **0.21 (0.06 to 0.35)** | **0.21 (0.07 to 0.34)** | **0.17 (0.03 to 0.32)** | **0.19 (0.04 to 0.34)** | **0.18 (0.03 to 0.34)** |
| High | **0.68 (0.45 to 0.91)** | **0.43 (0.18 to 0.67)** | **0.51 (0.28 to 0.73)** | **0.45 (0.22 to 0.68)** | **0.46 (0.21 to 0.70)** | **0.48 (0.16 to 0.79)** |
| Employment status |  |  |  |  |  |  |
| Economically active | **0.59 (0.46 to 0.71)** | **0.47 (0.33 to 0.61)** | **0.32 (0.17 to 0.46)** | **0.27 (0.12 to 0.42)** | **0.27 (0.12 to 0.42)** | 0.12 (-0.04 to 0.29) |
| Economically inactive | Reference | Reference | Reference | Reference | Reference | Reference |
| Wealth Index |  |  |  |  |  |  |
| Poorest and poor | Reference | Reference | Reference | Reference | Reference | Reference |
| Middle | 0.16 (-0.01 to 0.33) | 0.04 (-0.12 to 0.21) | 0.04 (-0.12 to 0.19) | -0.01 (-0.16 to 0.15) | 0.01 (-0.15 to 0.17) | 0.01 (-0.15 to 0.16) |
| Richer and richest | **0.25 (0.11 to 0.39)** | 0.03 (-0.11 to 0.18) | 0.07 (-0.06 to 0.21) | -0.02 (-0.15 to 0.12) | 0.01 (-0.13 to 0.15) | 0.03 (-0.11 to 0.16) |

CVHS –Cardiovascular health score; AHA – American heart association; ULR – Univariate linear regression; MLR – Multivariate linear regression.

* additionally significant interactions: Middle age x Middle educational level (-0.09 (-0.18 to -0.0)); Middle age x High educational level (-0.14 (-0.21 to -0.07)); Middle age x Economically active (0.11 (0.03 to 0.20)); Female x Middle educational level (0.13 (0.06 to 0.20)) Female x High educational level (0.08 (0.01 to 0.14)) and Female x Economically active (0.07 (0.01 to 0.14)).
